# Detection biomarkers of lung cancer using mini-GC-PID system integrated with micro GC column and micro pre-concentrator

**DOI:** 10.1186/1556-276X-9-576

**Published:** 2014-10-15

**Authors:** Jianhai Sun, Dafu Cui, Fengying Guan, Lulu Zhang, Xing Chen, Hui Li

**Affiliations:** 1 State Key Laboratory of Transducer Technology, Institute of Electronics, Chinese Academy of Sciences, Beijing 100190, China; 2 International Centre for Bamboo and Rattan, Key Laboratory of Bamboo and Rattan, Beijing 100102, China

**Keywords:** Micro gas chromatography column, Micro pre-concentrator, Mini-GC-PID system, Detection of biomarkers, Early diagnosis

## Abstract

The survival rate of lung cancer can be significantly improved by monitoring biomarkers in exhaled air that indicate diseases in early stage, so it is very important to develop micro analytical systems which can offer a fast, on-site, real-time detecting biomarkers in exhaled air. In this paper, a mini-gas chromatography (GC)-photo-ionization detector (PID) system integrated with a micro GC column and a micro pre-concentrator was developed for forming an inexpensive, fast, and non-invasive diagnostic tool for lung cancer. This system has very strong concentrate ability owing to its integrated micro pre-concentrator, which make the detection of trace components in exhaled air very easy. In addition, the integrated micro GC column can separate complex mixtures, which overcome low resolution and poor anti-interference ability of other instruments. The results indicated that the mini-GC-PID system can effectively separate and detect the biomarkers at parts-per-billion (ppb) level.

## Review

### Background

Lung cancer is the leading cause of death in the world today; its diagnosis very often happens late in the course of the disease since available diagnostic methods are not sufficiently sensitive and specific. So, early diagnosis is very important to significantly improve the survival rate of the patients. There are strong evidences [1-4] to suggest that lung cancer can be detected by molecular analysis of exhaled air in the early stage. Breath analysis represents a new diagnostic technique that is without risk for the patient; therefore, it is very important to develop microanalytical systems which can offer a fast, label-free, low cost, no damage and on-site monitoring biomarkers of lung cancer in exhaled air. In recent years, this research direction is under intensive research owing to its potential application, and numerous studies have focused on working on development of miniaturized instrumentations for detecting biomarkers of lung cancer in exhaled air.

Phillips et al. [1-3] had screened 22 VOC components (including benzene, toluene, ethylbenzene, styrene, pentane, nonane, decane, etc.) as biomarkers of lung cancer, and patients can be diagnosed by monitoring biomarkers in exhaled air. Gordon et al. [4] developed a nanoelectronic nose which can diagnose cancers and kidney failure by monitoring breath biomarkers, thus opening up a new approach for diagnosing diseases in the early stage.

At present, many efforts were performed to develop new approaches or mini instruments to diagnose lung cancer in the early stage. Gang et al. [5] diagnosed lung cancer in exhaled breath using gold nanoparticles. However, this method must be in combination with solid-phase microextraction (SPME) and gas chromatography (GC)/mass spectrometry, which makes the detection relatively slow and complex. Schwarz et al. [6] developed a PTR-MS to determine concentration patterns of volatile compounds in exhaled air. However, resolution of the detection is relatively low. Ligor et al. [7] determined volatile organic compounds appearing in exhaled air of lung cancer patients by SPME and gas chromatography/mass spectrometry, but SPME is a relatively insensitive method and compounds not observed in exhaled breath may be present at a concentration lower than the limit of detection (LOD). Olavi et al. [8] developed a laser spectroscopy to monitor exhaled air. However, the detectable components were very few (including HCN, NH_3_, C_2_H_2_, etc.). Many other works [9-17] also had played important contributions in early diagnosis of lung cancer by monitoring biomarkers in exhaled breath.

To fabricate an inexpensive, fast, and non-invasive diagnostic tool for lung cancer, in this paper, a mini-GC-photo-ionization detector (PID) system integrated with a micro GC column and a micro pre-concentrator was developed, and this system had very strong concentrate ability, which makes detection of trace components in exhaled air very easy. In addition, the integrated micro GC column can separate complex mixtures, which overcome low resolution and poor anti-interference ability of other instruments. The results indicated that the mini-GC-PID system can effectively separate and detect biomarkers at ppb level. The goal of this work was to present design, fabrication, and characterization of the mini-GC-PID system.

### Experimental details

#### ***Fabrication of micro pre-concentrator***

**Figure 1:**
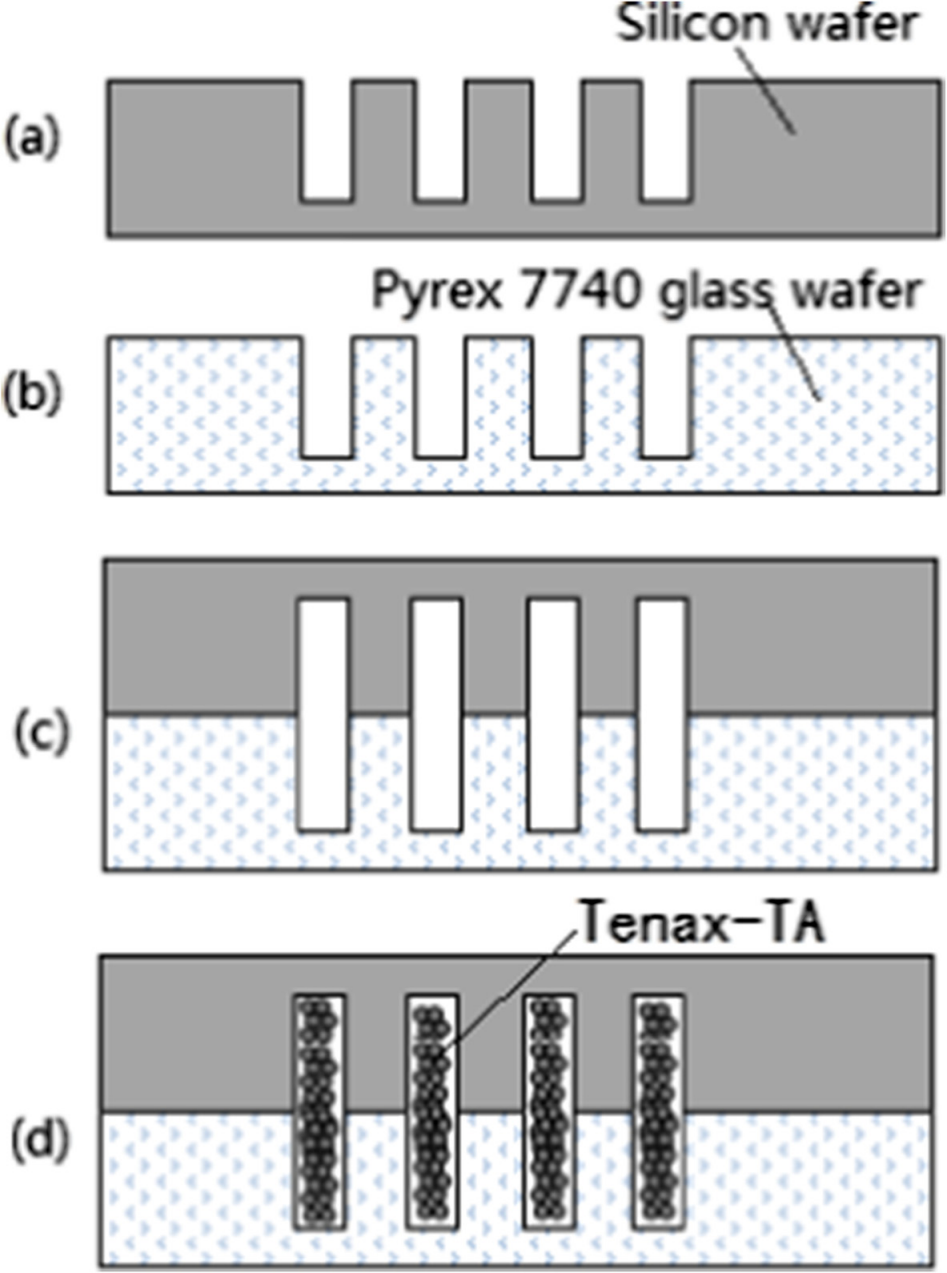
The process flow for fabrication of the pre-concentrator (a-d).

**Figure 2:**
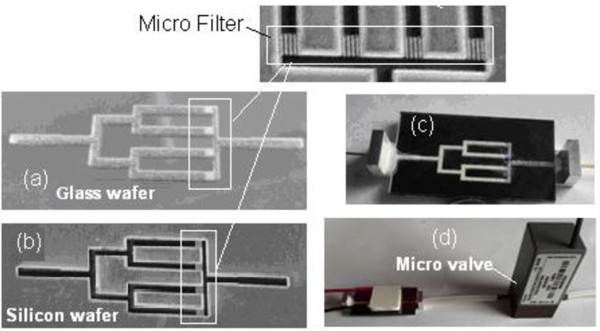
**Channels on wafers and images of different pre-concentrators and integrated micro valve.** Channels on glass **(a)** and silicon wafers **(b)** and images of fabricated pre-concentrator **(c)**, pre-concentrator, and integrated micro valve **(d)**.

As concentration of each component in exhaled air is very low (ranged from several parts per billion (ppb) to hundreds ppb) and the available diagnostic instruments are not sufficiently sensitive, the micro pre-concentrators developed for effectively concentrating components are very important and necessary, which significantly improve detection limit of mini-instruments with 1 ~ 2 orders. The micro pre-concentrator with reduced thermal mass can raise temperature much faster at lower power compared with conventional desorption tubes, realizing a much higher concentration factor. In this work, the proposed micro pre-concentrator filled with Tenax-TA was made by four parallel channels with a length from 20 mm and a cross section area of 1 mm × 0.8 mm. This choice was done on the basis of the consideration that four parallel channels ensure that the pre-concentrator can be filled with a sufficient amount of adsorbent materials (Tenax-TA powder), while a cross section area of 0.8 mm^2^ allows a simple cavitands introduction. The details of the fabrication process of the pre-concentrator are the following: Firstly, configuration of the micro pre-concentrator was drawn by AutoCAD software. Subsequently, rectangular micro channels were etched on silicon and glass wafer by a laser dicing saw according to the configuration (refer to Figure 1), and the length, depth, and width of the channels was 20 mm, 400 μm, and 1000 μm, respectively. Then these channels on silicon were aligned and bonded to these channels on glass wafer.In order to improve the concentration factor of the pre-concentrator, desorption of analyte must be fast and complete. Therefore, a micro heater which can rapidly increase temperature of pre-concentrator was fabricated on the backside of the micro pre-concentrator, leading to a fast thermal desorption of analyte. The heater was fabricated as a 20 nm/250 nm Cr/Pt stack deposited by a magnetron sputtering technology and patterned by a lift-off technology. The resistance of the heater was 8 Ω. The heater enables the temperature of the micro pre-concentrator to be increased at a speed of 10°C per second and the temperature could be raised up to 200°C in less than 30 s. Moreover, in order to increase adsorption capacity as much as possible in the adsorption process, a cooler was integrated on the upside of the pre-concentrator, and the temperature of the pre-concentrator can be cooled down to 5°C in 100 s. The heater and cooler cover the whole pre-concentrator chip region (i.e. the channels region) which have a temperature distribution over the chip surface as uniform as possible. In addition, in order to compress peak broadening and further improve the concentration factor, a micro valve (purchased from Shenzhen Keyto Fluid Control Co., Ltd, Shenzhen, China) was integrated beyond the pre-concentrator, which can close the released components during the thermal desorption process, thus increasing the concentration factor as maximum as possible. Images of the fabricated micro pre-concentrator are shown in Figure 2, in which the micro filters can prevent the Tenax-TA powder out of the pre-concentrator from the outlet.

Then, one end of the pre-concentrator was connected with a capillary which was emerged into the Tenax-TA powder, and the other end of the pre-concentrator was connected with a micro pump. After these channels were filled with Tenax-TA powder (80 to 100 mesh), the pre-concentrator was put into a temperature programmed oven under a nitrogen flow inside. The temperature of the oven was firstly increased gradually by 5°C/min until 200°C, and then the temperature of the oven was kept at 200°C for 4 h.

#### ***Fabrication of micro dryer and purifier***

**Figure 3:**
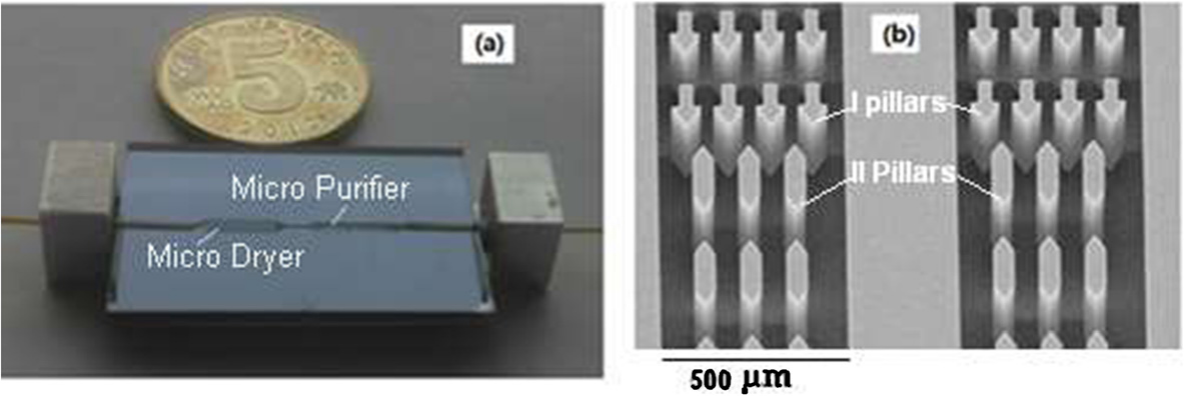
The fabricated micro dryer and purifier (a) and micro-pillars in channel (b).

As the vapor can make the GC column inactive, a micro dryer and purifier were integrated into the mini-GC-PID system for removing vapor in exhaled air. In configuration of the micro dryer, two types of micro-pillars were arranged in the channel, the former (I pillar) was used to rapidly atomize droplets, and the latter (II pillar) was applied for rapid vaporization. The details of the fabrication process of the micro dryer and purifier are the following: Firstly, a layer of 300-nm silicon nitride was deposited by a low-pressure chemical vapor deposition technology on a (100) n-type low-resistivity silicon wafer with a thickness of 550 μm and a diameter of 76 mm, and then a layer of 200 nm aluminum film was deposited by an electron-beam evaporation technology on top of silicon nitride layer, served as a mask for silicon etching. Secondly, a layer of 2-μm photoresist was coated on the wafer and patterned as a mask for aluminum etching. Subsequently, aluminum without protection of the photoresist was etched away by a H_3_PO_4_ etching agent with silicon nitride surface exposed. Then, a reactive-ion etching technology was used to etch the silicon nitride and a deep reactive-ion etching was conducted to form rectangular micro channel and micro-pillars. In order to rapidly increase temperature of the micro dryer, a micro heater was fabricated on the backside of the micro dryer. The heater was fabricated as a 20 nm/250 nm Cr/Pt stack deposited by a magnetron sputtering technology and patterned by a lift-off technology, and resistance of the heater was 8 Ω. The heater enables temperature of the micro dryer to be increased at a speed of 10°C/s and the temperature could be raised up to 200°C in less than 30 s. Then, a Pyrex 7740 glass wafer with a thickness of 1 mm and a diameter of 76 mm was bonded to the silicon wafer. In addition, to remove vapor, molecular sieve 5A (60 to 80 mesh) was filled in the channel of the micro purifier. The length, depth, and width of the channel was 10 mm, 400 μm, and 500 μm, respectively. Figure 3 shows the fabricated micro dryer and purifier.

#### ***Fabrication of micro GC column***

**Figure 4:**
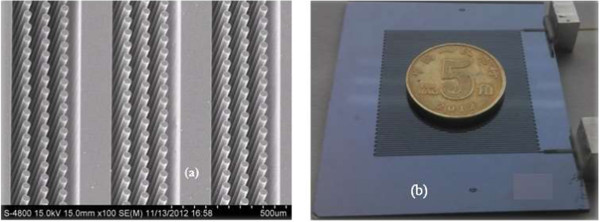
The embedded micro-pillars in channels (a) and photograph of the fabricated GC column (b).

In order to separate biomarkers of lung cancer in exhaled air, a micro GC column coated with OV-101 stationary phase was integrated into mini-GC-PID system. The micro GC column embedded micro-pillars [18] was developed for increasing overall surface area of the column which is able to support more of the stationary phase and reduce effective width of the column, thus demonstrating higher separation efficiency. In order to reduce volume of the GC system and rapidly increase temperature of the column, micro heaters were fabricated on backside of the micro GC column, and the heaters enable the temperature to be increased at a speed of 2°C/s and the temperature could be raised up to 200°C in less than 2 min. The fabricated micro GC column is shown in Figure 4.

#### ***The integrated mini-GC-PID system***

**Figure 5:**
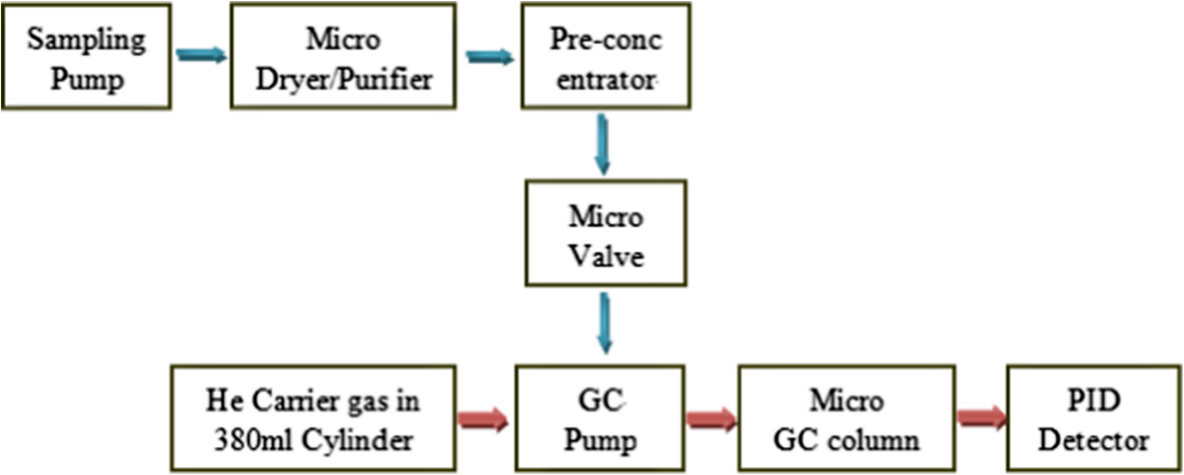
A schematic representation of the mini-GC-PID system architecture.

In this paper, the mini-GC-PID system integrated with a micro dryer and purifier, a micro pre-concentrator, a micro GC column, and a mini-PID detector [19] was developed for detecting biomarkers that indicate diseases in the early stage. The mini-PID with a volume of 10 μl smaller than its commercial PID counterparts demonstrated a detection limit of 1 ppb (benzene acted as the subject matter), moreover, the optimal average carrier gas velocity (only ranged from 1 to 5 sccm) is very small owing to the very small size of the ionization chamber. Figure 5 shows a schematic representation of the mini-GC-PID system architecture. The mini-GC-PID system is characterized by a series of innovations. Firstly, the concentration factor of the pre-concentrator was about 15, which can improve detection limit of GC-PID system with 1 to 2 orders, and makes the system easy to deploy and suitable for in-field use; Secondly, a micro GC column instead of a conventional column was used to separate mixtures, which can greatly reduce volume of the system; In addition, the mini-GC-PID system can process more complex samples owing to its integrated micro dryer, purifier, and pre-concentrator.

#### ***Materials and reagents***

In this paper, the biomarkers of lung cancer were detected using the proposed mini-GC-PID system. The pure H_e_ was used as carrier gas, sample I (provided by the Beijing Hua Yuan Gas Chemical Industry Co., Ltd, Beijing, China) was composed of benzene with a concentration of 5 ppm, and sample II (provided by the Beijing Hua Yuan Gas Chemical Industry Co., Ltd) was composed of 7 components (concentrations of benzene, toluene, ethylbenzene, styrene, pentane, nonane, and decane are 10, 7, 6, 10, 5, 8, and 6 ppm, respectively). Tenax-TA and OV-101 were purchased from Sigma-Aldrich (St. Louis, MO, USA). In order to accurately simulate the actual samples, these standard samples were diluted 100 times, thus making concentration of these standard samples close to the actual samples.

### Results and discussion

#### ***The characterization of the micro pre-concentrator***

**Figure 6:**
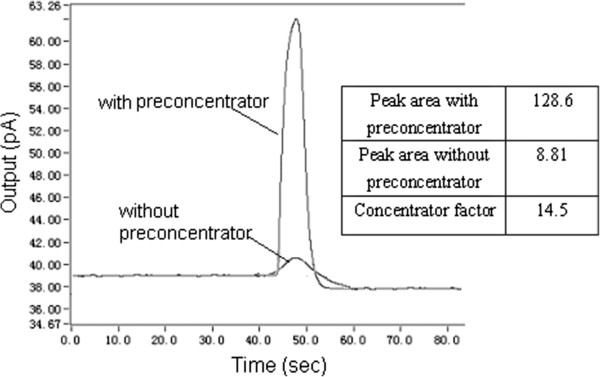
The performance of the pre-concentrator.

In order to evaluate capabilities of the micro pre-concentrator, the diluted sample was concentrated. The details of the experiment were reported as follows: Initially, one end of the micro pre-concentrator was connected with a sampling pump and the other end was connected with a micro valve. The sampling pump had taken an amount of the diluted sample I and delivered it into the micro pre-concentrator which was in cooling state. Then, the valve was closed and the micro pre-concentrator was heated, after the adsorbed aromatic benzene was completely released, the micro valve was opened and the released component was consequently delivered into the PID by carrier gas. Figure 6 indicates the comparison of PID with and without the fabricated pre-concentrator response to the diluted sample I. The result shows that the developed pre-concentrator can effectively concentrate the diluted component about 15 times. Moreover, the chromatography peak broadening was greatly compressed.

#### ***Rapid detection of biomarkers***

**Figure 7:**
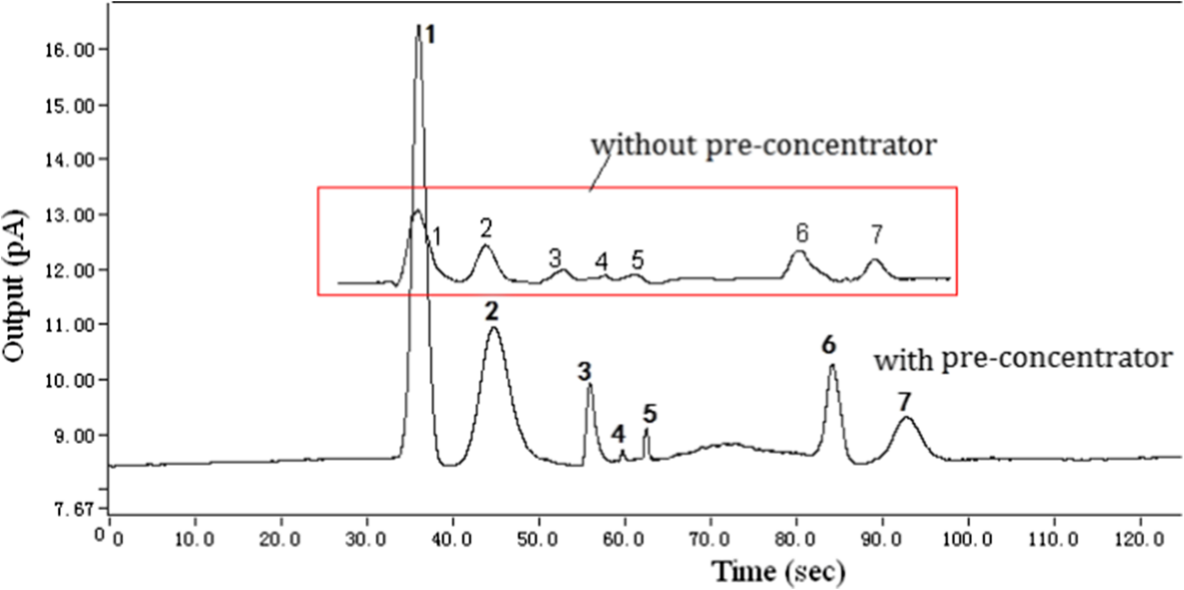
**Chromatogram of the diluted sample II.** Compound identification in order of elution: (1) benzene, (2) toluene, (3) ethylbenzene, (4) styrene, (5) pentane, (6) nonane, (7) decane.

In order to evaluate capabilities of the proposed mini GC-PID system for separating and detecting biomarkers that indicates diseases in the early stage of lung cancer, the experiments were performed under isothermal conditions at 40°C with a carrier gas linear velocity of 18 cm/s (or the volume flow rate of 1.2 sccm). The diluted sample II was delivered into the micro dryer and micro pre-concentrator by a sampling pump. After concentrated by the pre-concentrator, these released components during thermal desorption process entered into the GC column by opening the valve and then were rapidly separated and detected by the micro column and mini-PID, respectively. In order to demonstrate the advantage of the pre-concentrator, the experiment performed without the pre-concentrator was used as a control. Figure 7 shows chromatograms of the diluted sample II performed with and without pre-concentrator, and Table 1 indicates the comparison of the peak area calculated from these chromatograms. As we can see from the result, these components can be effectively concentrated by the micro pre-concentrator. Moreover, these released components rapidly entered into the micro GC column after opening the micro valve, and these components were completely separated and effectively detected in less than 100 s. In addition, peak broadening and peak tailing of the chromatogram obtained from the mini-PID system integrated with a micro pre-concentrator was smaller than that of chromatogram performed without the micro pre-concentrator. Though the chromatographic peaks of styrene and pentane were relatively small, we can still clearly see their peaks, however, the chromatographic peak of styrene can hardly be observed in the chromatogram performed without the micro pre-concentrator.

**Table 1:** The comparison of the peak area calculated from these chromatograms

	**Benzene**	**Toluene**	**Ethylbenzene**	**Styrene**	**Pentane**	**Nonane**	**Decane**
Peak area with pre-concentrator	18.072	12.726	1.807	0.151	0.376	4.066	3.050
Peak area without pre-concentrator	2.048	1.355	0.226	-	-	0.750	0.338
Concentrator factor	8.8	9.4	8	-	-	5.5	9

Because cooperation with hospital is still underway, actual samples were difficult to obtain. Monitoring and detecting the actual patient samples will be performed in the future. However, the above results still demonstrated the mini-GC-PID system was able to effectively detect the biomarkers in the exhaled air that indicates diseases in the early stage.

## Conclusions

The work here demonstrates that it is possible to fabricate a mini-GC-PID system integrated with a micro pre-concentrator and a micro GC column. Based on above experimental results, this system has strong concentrate ability and overcomes low resolution and poor anti-interference ability of other instruments owing to its integrated micro pre-concentrator and micro GC column. The results also indicated that the mini-GC-PID system effectively separated and detected the biomarkers at ppb level. However, the standard sample instead of the actual sample was used for performing the experiments due to absence of actual sample. But the work will be performed in the future and the results will be reported in next works.

## Competing interests

The authors declare that they have no competing interests.

## Authors’ contributions

JHS carried out the fabrication of micro dryer, purifier, pre-concentrator, and GC column, participated in the design of the study, and drafted the manuscript. DFC helped to fabricate the GC-PID system. FYG participated in the design of the study and helped to draft the manuscript. LLZ and XC helped to draft the manuscript. HL participated in the design of the study and helped to draft the manuscript. All authors read and approved the final manuscript.
